# Ophthalmology and Otology

**Published:** 1876-10

**Authors:** 


### VIII. Ophthalmology and Otology.

1. *The Injurious Effects of the Nasal Douche.* Dr. Henry L. Shaw. (*Boston Med. and Surg. Journal*, 1876, No. 23.)

For many years aurists have considered the treatment of nasal catarrh with the douche as attended with serious danger, this danger being the flooding of the Eustachian tubes and passing of liquid into the tympanum, thereby causing inflammation of that organ with all its sequelæ. As early as 1869 Dr. Roosa speaks of the danger of the douche, and in an article published in the "Archives of Ophthalm. and Otology," Vol. II., he has collected from various sources sixteen cases in which results more or less serious followed its use. Dr. Pardee (in *New York Med. Gaz.*, Vol. VI.) reports several cases in which acute aural inflammation was caused by it. Other cases have been reported, the ætiology of which cannot be doubted, all tending to show its injurious effects. The opinion that the employment of the douche should be discountenanced by the profession, is now quite generally accepted by aurists. Dr. S., however, is inclined to believe that the use of the nasal syringe, also, and all other appliances for *flooding* the nasal cavity, is attended with some risk, and that it is not even necessary for liquids to enter the tympanum to produce harm. In support of this opinion eighteen cases of injury are presented, in sixteen of which it was from the nasal douche and syringe, in one from the forcing of liquid into the tympanum by the Valsavian method, and in one from the snuffing of liquid into the nostrils. In five cases there was acute otitis media, with perforation of the drum-head, from the douche. In five there was acute otitis media, without perforation of the drum-head, from the douche. In two there was subacute otitis media, without perforation of the drum-head, from the douche. In one there was increase of chronic otitis media from the douche. In one there was acute otitis media, without perforation of the drum-head, from the syringe. In one there was subacute otitis media, without perforation of the drum-head, from the syringe. In one there was acute otitis media, with perforation of the drum-head, from snuffing liquids into the nostrils. In one there was subacute otitis media in one ear, and formation of polypus in the other ear, from forcing liquids into the tympanum by the Valsavian method.

2. *Artificial Atrophy of the Eyeball.* S. VIDOR AND N. FEUER. (*Memorabil.*, xxi., 2.)

Græfe, we believe, was the first who suggested, in certain deformities of the eye, the attempt to induce a partial and gradual shrinkage of the eyeball by passing a thread through the posterior half of the eyeball. This thread is left in situ until the necessary inflammation in the tissues of the eye is started, to insure the subsequent atrophy. The final object of this procedure is the obtaining of a serviceable stump which is better adapted for the support of an artificial eye than the socket which is left after the enucleation of the eyeball.

Vidor (*Jahrb. f. Kinderheilk*) reports three such cases. The first case was an hydrophthalmus in a boy five years old. The globe was greatly expanded in all directions; the cornea was hazy and the sclerotic was very thin; cause, unknown; perception of light, none. A thread was put through the eyeball behind the ciliary ring and from one side of the sclerotic to the other. But for twelve days the presence of the thread was so well tolerated that the doctor moved it to and fro several times in order to produce more irritation. Three days later pus appeared behind the cornea; the thread was removed; a copious discharge of pus took place, and from the seventh day after removing the thread the suppuration subsided. Within one month the eyeball had shrunken to half the normal size, and was free of all irritation.

In the second case (staphyloma of cornea and sclerotic) the thread remained in the eyeball for seven full weeks without producing any violent reaction. The eyeball, however, began to get softer and smaller after the fifth day, and finally the thread had to be withdrawn for fear the ball would shrink too much.

The third eye also showed a great indifference for the thread, and did not secrete any pus until the thread had been pulled upon intentionally; still it had to remain in the eye six weeks.

Compared with these observations, Feuer's nine cases (*Pester med. chir. Presse*) are interesting for the short time during which the thread remained in the eyeball; the longest period being thirty-six hours, and the shortest four hours. The thread was removed as soon as the ocular conjunctiva became œdematous.

In three cases the reaction was moderate, and the globe shrank to the desired degree. In the other cases the reaction was more violent, and in some the inflammation induced by the thread assumed the character of a true panophthalmitis, though the thread had been left in the eye but a few (5, 10, 13) hours.

